# Long non-coding RNAs in oncourology

**DOI:** 10.1016/j.ncrna.2021.08.001

**Published:** 2021-08-26

**Authors:** Ilgiz Gareev, Yulia Gileva, Aleksandr Dzidzaria, Ozal Beylerli, Valentin Pavlov, Murad Agaverdiev, Bakhodur Mazorov, Ilfat Biganyakov, Andranik Vardikyan, Mei Jin, Aamir Ahmad

**Affiliations:** a Bashkir State Medical University, Ufa, Republic of Bashkortostan, 450008, Russia; b Urology Department, Russian Scientific Center of Radiology of the Ministry of Health of the Russian Federation, Moscow, Russia; c The First Affiliated Hospital of Harbin Medical University, 23 Youzheng St, Harbin, 150001, Heilongjiang Province, China; d Interim Translational Research Institute, Academic Health System, Hamad Medical Corporation, Doha, Qatar

**Keywords:** Long non-coding RNAs, Oncourology, Methods, Tumor, Expression, Biomarker, Targets

## Abstract

For several decades, research in tumor biology has focused on the involvement of genes encoding a protein. Only recently has it been discovered that a whole class of molecules called non-coding RNAs (ncRNAs) play a key regulatory role in health and disease. Long noncoding RNAs (lncRNAs) are a group of noncoding RNAs longer than 200 nucleotides. It has been found that lncRNAs play a fundamental role in the biology of many types of tumors, including tumors of the genitourinary system. As a result, hundreds of clinical trials dedicated to oncourology have begun, using lncRNA as new biomarkers or treatments. Identifying new specific biomarkers, in the form of lncRNAs, will increase the ability to differentiate the tumor and other processes, determine the localization and extent of the tumor, and the ability to predict the course of the disease, and plan treatment. Therapy of tumors, especially malignant ones, is also a difficult task. When surgery and chemotherapy fail, radiation therapy becomes the treatment choice. Therefore, the possibility that lncRNAs could represent innovative therapeutic agents or targets is an exciting idea. However, the possibility of their use in modern clinical practice is limited, and this is associated with several problems at the pre-, analytical and post-analytical stages. Another problem in the study of lncRNAs is the large number and variety of their functions in tumors. Therefore, solving technological problems in lncRNAs study in oncourology may open up new possibilities for lncRNAs use in modern clinical practice.

## 1 Introduction

Tumors are one of the leading causes of death in the world, second only to cardiovascular diseases [1]. They represent an extensive and diverse class of diseases that manifest themselves in various forms, with varying degrees of severity and varying responses to treatment. Over the past several decades, certain success has been achieved in studies of the pathological and molecular mechanisms of the emergence and development of tumors. The study of pathogenesis is necessary in the search for new strategies for the diagnosis, prognosis and treatment of this deadly disease [2]. Oncourology occupy a special place in oncology. Despite advances in the treatment of these malignancies, urologists still face the challenge of improving diagnosis in the early or “pre-early” stages of the disease by developing methods that can detect neoplasms, bypassing the side effects of biopsy and other traditional diagnostic approaches [2]. In this regard, extracellular molecules in biological fluids embedded in microvesicles or protected by RNA-binding proteins/lipids are likely to provide diagnostic, prognostic, or even therapeutic targets in the fight against this type of tumor.

Non-coding RNAs (ncRNAs) refers to RNA molecules that are not translated, that is, proteins are not synthesized by their sequence. NcRNAs encompass a huge number of RNA classes and perform a wide range of biological functions, such as regulating gene expression, protecting the genome from exogenous DNA, and controlling DNA synthesis [3]. One of these classes of ncRNAs that are widely studied are long non-coding RNAs (lncRNAs). LncRNAs are a group of ncRNAs over 200 nucleotides in length. Because of their length, lncRNAs nucleotide chains have the unique ability to accept many complex secondary and tertiary structures, allowing them to perform specific functions necessary for the existence of an organism. LncRNAs cannot encode protein, but they can modulate gene expression at epigenetic (eg, DNA methylation, histone modification), transcriptional (eg, recruitment of transcription factors) and post-transcriptional (eg, regulation of microRNA (miRNA) and messenger RNA (mRNA) stability) levels [4]. An increasing number of studies have demonstrated deregulation or aberrant expression of lncRNAs in various types of human diseases [5]. Many studies have also shown aberrant lncRNAs expression in various tumors, as in tumors of the genitourinary system, which was directly associated with carcinogenesis, metastasis and disease stages (Table 1) [[6], [7], [8], [9], [10], [11], [12]]. Moreover, lncRNAs can be found in human biological fluids such as blood and urine [13]. The so-called circulating lncRNAs can be secreted from tumor cells into human biological fluids in extracellular vesicles (EVs), like microvesicles or exosomes. Such lncRNAs are resistant to the effects of RNases, which makes them attractive as new diagnostic and prognostic non-invasive biomarkers [13]. It has been proven that exosomes play an important role in intercellular communication. Exosomes, in addition to transferring lncRNAs, are used to transfer other information like DNA, proteins, etc. from one cell to another, and tumor cells play a particularly important role in the production of exosomes [14]. Now, many promising studies have studied circulating lncRNAs as non-invasive biomarkers in tumors of the genitourinary system (Table 2) [[15], [16], [17], [18], [19], [20]]. As an example, lncRNA prostate cancer antigen 3 (PCA3), which is the most striking example of the use of lncRNA as a non-invasive biomarker in prostate cancer (PC), approved by the FDA (Food and Drug Administration) [15].

**Table 1:** LncRNAs involved in carcinogenesis of tumors of the genitourinary system.

LncRNA	Type of tumor	Expression	Gene-Targets	Biological function	Ref.
MEG3	Clear cell renal cell carcinoma	Decreased	Bcl-2, procaspase-9	Tumor suppressor. Promotes apoptosis of tumor cells.	[6]
MALAT1	Clear cell renal cell carcinoma	Increased	miR-200s, ZEB2	Oncogenic properties. Promotes proliferation, migration and invasion of tumor cells	[7]
GAS6-AS2	Bladder cancer	Increased	miR-298, CDK9	Oncogenic properties. Promotes proliferation and metastasis	[8]
CASC11	Bladder cancer	Increased	miR-150	Oncogenic properties. Promotes proliferation, migration and invasion of tumor cells	[9]
CCAT1	Prostate cancer	Increased	DDX5, mIR-28-5p	Oncogenic properties. Promotes the proliferation of tumor cells	[10]
HOXD-AS1	Castration-resistant prostate cancer	Increased	WDR5	Oncogenic properties. Promotes proliferation, castration resistance and chemoresistance	[11]
XIST	Prostate cancer	Decreased	RKIP, miR-23a	Tumor suppressor. Suppression of tumor growth and progression	[12]

**Abbreviations:** LncRNA, Long non-coding RNA; miRNA, microRNA; MEG3, Maternally expressed 3; MALAT1, Metastasis associated lung adenocarcinoma transcript 1; GAS6-AS2, GAS6 antisense RNA 2; CASC11, Cancer susceptibility 11; CCAT1, Colon cancer associated transcript 1; HOXD-AS1, HOXD cluster antisense RNA 1; XIST, X-inactive specific transcript; Bcl-2, B-cell lymphoma 2; ZEB2, Zinc finger E-box binding homeobox 2; CDK9, Cyclin-dependent kinase 9; DDX5, DEAD-Box Helicase 5; WDR5, WD Repeat Domain 5; RKIP, Raf kinase inhibitory protein.

**Table 2:** Validity of circulating lnсRNAs as biomarkers for diagnosis and prognosis of tumors of the genitourinary system. Exemplificative data from most recently published studies is presented. AUC ≥0, 75 is considered diagnostically significant for the biomarker. Kaplan–Meier curves and log-rank tests were used in articles to evaluate the prognostic significance of circulating lnсRNAs./, not mentioned in the article.

LncRNA	Type of tumors	Sample type	Expression	Diagnosis	Prognosis	Specificity %	Sensitivity %	AUC	References
PCA3	PC	Urine	Increased	Yes	Yes	60.1	94.9	0.87	[15]
MALAT 1	PC	Plasma	Increased	Yes	No	58.6	84.8	0.84	[16]
TUC338	BC	Plasma	Increased	Yes	No	/	/	0.92	[17]
Exosomal UBC1	BC	Serum	Increased	Yes	Yes	/	/	0.75	[18]
Exosomal H19	BC	Serum	Increased	Yes	Yes	84.6	67.3	0.76	[19]
LET, PVT1, PANDAR, PTENP1, and linc00963	CCRCC	Serum	Decreased	Yes	No	/	/	Comb. 0.9	[20]

**Abbreviations:** LncRNA, Long non-coding RNA; MALAT1, Metastasis associated lung adenocarcinoma transcript 1; PCA3, Prostate cancer antigen 3; AUC, Area under the Curve; TUC338, Transcribed ultra-conserved region 338; UBC1, Upregulated in bladder cancer 1; PVT1, Plasmacytoma variant translocation 1; PANDAR, Promoter Of CDKN1A Antisense DNA Damage Activated RNA; PC, Prostate cancer; BC, Bladder cancer; CCRCC, Clear cell renal cell carcinoma.

Aberrant expression of lncRNAs in tumor cells is a hallmark of a tumor, and lncRNAs may play a role as suppressors of tumor genes or oncogenes, depending on the cellular context and different functions of the target genes. Tumor lncRNAs have certain properties of oncogenes that can specifically control the regulation of the expression of tumor suppressor genes with subsequent inhibition of their expression. Thus, indirectly promote the development of the tumor. In addition, such lncRNAs are mainly overexpressed in the tumor cell. In other words, their expression level increases in the case of neoplastic transformation of normal cells [13,14]. Antitumor lncRNAs can specifically control the regulation of the expression of oncogenes and inhibit their expression in tumors. Therefore, they can inhibit the transcription of target oncogenes and ultimately inhibit tumor growth or genesis. In contrast to the first, the level of expression of such lncRNAs in the tumor cell is reduced [14]. The study of lncRNAs in oncourology is one of the most promising directions in modern fundamental research and can help in the discovery of a significant number of new potential targets for new generation targeted drugs, as well as for the development of gene therapy. In this review, we will consider the problems and ways of their possible solution related to the profiling of lncRNAs expression in oncourology.

## 2 Methodological aspects of lncRNA expression profiling

### 2.1 Pre-analytical, analytical and post-analytical stages

**Fig. 1:**
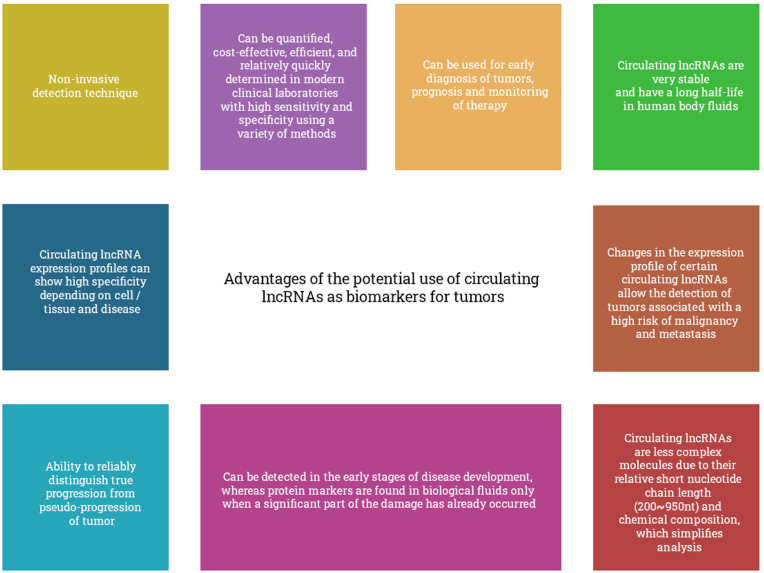
Advantages of the potential use of circulating lncRNAs as biomarkers for tumors.

With early detection of a tumor, there is a high probability of positive results with effective treatment. Early diagnosis leads to more effective therapy, as a result of which the duration and quality of life of patients are increased. Therefore, the search for reliable, accurate, and non-invasive biomarkers for early diagnosis, prognosis, and monitoring of tumor treatment is an acute problem to this day. Research on the study of DNA or RNA molecules as potential biomarkers is not innovative, but, unfortunately, despite this, their results do not correspond to their use in modern clinical practice. Profiling of lncRNA expression in samples of cells and tissues, as well as in biological fluids of the human body, is feasible using various methods [21]. Proteins as biomarkers are sensitive indicators of physiological and pathological processes, including tumors, as well as responses to therapy. However, in turn, proteins are inferior to lncRNA for several reasons (Fig. 1) [22,23]. An ideal biomarker should be easy to detect, requiring a simple measurement methodology. However, in the case of circulating lncRNA, some problems arise at the pre-analytical, analytical, and post-analytical stages, the need to solve which is an important task for the possible introduction of circulating lncRNA as non-invasive biomarkers into clinical practice.

Another factor limiting the use of circulating lncRNAs as a diagnostic tool in oncourology is associated with the fact that the same lncRNAs reported as potential biomarkers are found in patients with different types of tumors. For example, circulating metastasis associated lung adenocarcinoma transcript 1 (MALAT1) has been found in overexpressed serum in patients with PC, bladder cancer (BC), and renal cell carcinoma (RCC). Moreover, the results are inconsistent even among very similar studies with the same diseases [23,24].

Reduced expression of endogenous lncRNA in tumor tissues may be the result of genetic changes or mechanisms of epigenetic regulation, but a decrease in the level of expression of circulating lncRNA in biological fluids can occur only if the tumor itself negatively affects the expression of endogenous lncRNA in other cells or reduces their stability. In other words, a decrease in the level of expression of circulating lncRNA in biological fluids can be attributed to nonspecific responses to the presence of a tumor [25]. In addition, the presence of a tumor cannot be correlated with the activation of these lncRNAs. Due to the anticoagulant system of the blood, only a small number of tumors, such as advanced tumors, resulting in overexpression of specific circulating lncRNAs in the blood. Accordingly, it is more likely that the detection of circulating lncRNA in biological fluids is also the result of the response (s) to the presence of a tumor in the body [26].

One of the important properties of an ideal biomarker is its stability in a biological environment. There were opinions that lncRNAs are less stable in human tissues and biological fluids, and are more susceptible to degradation than miRNAs, which may have been caused by their nucleotide length. However, Kraus et al. showed that some lncRNAs (lncRNA LUCA-15-specific transcript (LUST), lncRNA IGF2-AS (IGF2AS), lncRNA-7SK, lncRNA HOXA6as, lncRNA neuroblastoma differentiation marker 29 (NDM29)) in healthy brain tissue are even more stable than miRNAs [27]. Other researchers also indicated that intragenic and *cis*-antisense lncRNAs (half-life more than 16 h) are more stable than most lncRNAs that form from intron regions within a gene [28]. The specific half-life of lncRNA depends not only on its coding site in genome modifications but also on subcellular localization and function [29]. Moreover, the presence of some circulating lncRNAs, such as H19, in plasma confirms the high stability during incubation of plasma in ethylenediaminetetraacetic acid (EDTA) tube overnight at room temperature [30].

The next question concerns the standardization of the total RNA isolation method. There are drawbacks to specific sampling and storage methods. Tumor tissue can be compared with healthy control tissue samples from the same patient or with a history of healthy donor samples. In our opinion, healthy tissue samples from the studied tumor patients are not the best standards for standardization due to the risk of false results. There are also no reference methods for the isolation of total RNA, including its fractions. Total RNA from tissues and cell cultures is usually isolated using standard methods for total RNA isolation based on classical methods such as guanidine thiocyanate-phenol-chloroform extraction of total RNA or newer ones based on a column system with silica [31]. However, the silica column approach appears to be better than guanidine thiocyanate-phenol-chloroform total RNA extraction, especially in the case of extraction of circulating lncRNA from human biological fluids [32].

For circulating lncRNAs, the choice of the type of sample (biological fluid), their processing, as well as in the case of blood, the presence of hemolyzed cells in plasma/serum, can affect the preparation of the sample for further testing and ultimately the results. Due to coagulation and hemolysis, blood cells release endogenous lncRNA into plasma or serum, which can distort the results of the work performed on profiling the expression of circulating lncRNA [33]. However, the use of blood collection vacutainers can minimize background RNA levels and eliminate false results in quantification [34]. Because of the constant contact of urinary tract tumors with urine, circulating lncRNAs from urine is an important source of non-invasive biomarkers. Various pre-analytical factors affect the collection of urine samples and the measurement of lncRNA in general, native urine, or, after centrifugation, in sediments and/or supernatants [35]. For prostate cancer with urine-based circulating lncRNA studies, it is very important whether the prostate was massaged or palpated prior to urine collection. Collecting urine after digital rectal examination it is recommended to prepare urine samples for lncRNA PCA3 determination using the PROGENSA PCA3 kit (Hologic Gene-Probe Inc., San Diego, Calif.) [36]. Urine samples are collected in prepared tubes containing a stabilizing reagent. This processing process was developed after comparative studies to determine the process with the highest diagnostic accuracy for this marker. This manipulation is currently commonly used in all studies of PC markers, either to measure the total, native urine, or its sediments or supernatants. Norgen Biotek also offers kits that allow you to concentrate preserve and isolate lncRNAs in urine with the stability of up to 2 years at room temperature [8].

Another very important issue is the definition of qualitative and quantitative indicators. Using the NanoDrop-2000 spectrophotometer, you can determine the quantitative and qualitative indicators of total RNA. Biological fluids, in contrast to tissue or cell cultures, have very low total RNA concentrations (in the range of 15–100 ng/μl). Qualitative indicators are determined by the absorption spectrum of the resulting solution with total RNA at three wavelengths 230, 260, 280 nm. According to the absorption spectrum of a total RNA solution, the degree of RNA purification from proteins, polysaccharides, guanidine, phenol, and EDTA is approximately estimated. The absorption ratio at 260/280 nm should be approximately equal to 2 (1.9–2.1); if the absorption ratio was less than 1.9, then additional purification of total RNA from proteins and guanidine thiocyanate is carried out. If this ratio is greatly increased, this indicates the decay of total RNA. The absorption ratio at 260/230 nm should be slightly over 2 (2–2.3). If this ratio is less than the established value, then the sample is contaminated with phenol, polysaccharides, or EDTA and requires mandatory cleaning, since these substances inhibit many enzymatic reactions, including polymerase chain reaction (PCR) [32,37].

Using the correct housekeeping gene to normalize data remains problematic in measuring circulating lncRNA expression. In the absence of such genes, most studies with circulating lncRNA are based on other lncRNAs [36]. Inappropriate advice affects results and makes it difficult to compare different studies. Normalization problems have been observed in the case of expression studies with circulating miRNAs [38]. Small nucleolar RNAs (snRNAs) such as U6, used as data normalization for circulating miRNAs, show low expression due to instability in biological fluids. This situation suggests that proper normalization is an important step in presenting and comparing data. It should also be checked whether specific housekeeping genes are needed for both endogenous and circulating lncRNAs. For example, there are specific “housekeeping” genes in studies of changes in the expression of endogenous lncRNA, which are suitable only for studies with normal tissues [39]. Some can be used as universal housekeeping genes in profiling lncRNA expression in various types of tumor tissues, such as glyceraldehyde 3-phosphate dehydrogenase (GAPDH). Thus, it should be checked whether different types of housekeeping genes are needed to specifically associate lncRNA with tumors. In their study, Fang et al. Tested 16 different housekeeping genes for tumor, metastatic and normal cells (Actin Beta (ACTB), Tubulin alpha-3 chain (TUBA3), tubulin K-alpha-1, GAPDH, or Beta-2-Microglobulin (B2M)); for example, ACTB was chosen as the best housekeeping gene for MALAT1 [40]. Dong et al. conducted a meta-analysis on the potential use of ACTB, GAPDH, hypoxanthine–guanine phosphoribosyltransferase (HPRT), 18S RNA, cycle gene (CYC), and glucuronidase beta (GUSB) as housekeeping genes to normalize circulating lncRNA data in the serum of cancer patients, and ACTB was selected as the best gene. Moreover, ACTB is stable after temperature changes in serum samples [41].

### 2.2 Expression profiling methods

One of the main questions for the quantitative measurement of the level of lncRNA expression is the choice of the platform itself, i.e. expression profile analysis method. Common methods for analyzing the expression profile for lncRNA are real-time polymerase chain reaction (real-time PCR), microarrays, and next generation sequencing (NGS) [42]. Profiling hundreds of differentially expressed circulating lncRNAs from biological fluids is a technical challenge. Determination of the expression profile of circulating lncRNA by real-time PCR can be performed by absolute or relative method. Real-time PCR is considered a reference method [43]. The advantage is that it is easy to use in daily practice. In addition, it is sensitive, specific and offers a wide measurement range. Using this technology, expression can be profiled individually for a specific lncRNA or in panels of several hundred lncRNAs. The real-time PCR method used in lncRNA expression profiling studies is based on the use of dye probes such as SYBR-Green. Real-time PCR analysis requires the correct choice of a method for the synthesis of complementary DNA (cDNA) on a reverse transcription PCR (RT-PCR) platform and housekeeping genes for data normalization [44]. There are no standard methods for reverse transcription of lncRNA. Some lncRNAs have endogenous poly (A) tails, but others do not. Moreover, most lncRNAs are present in low copy numbers, making it difficult to quantify with conventional methods. These lncRNAs require the addition of oligonucleotide primers prior to cDNA synthesis. This approach makes it possible to increase the specificity and sensitivity of the quantitative assessment of both endogenous and circulating lncRNA [45]. However, in most studies, cDNA is obtained using various commercial kits that already contain mixtures of oligonucleotide or random hexameric primers [7,15].

Microarrays represent a miniature hybridization system that allows simultaneous analysis of several hundred lncRNAs at a high level. This method makes it possible to carry out combinatorial analysis between lncRNA and gene expression on the same sample, which allows us to study the function of lncRNA and target genes [46]. However, the efficiency of lncRNA isolation from biological fluids is much lower than from cells or tissues, and microarrays do not actually represent a method of quantitative analysis. Therefore, microarrays should be used for initial screening, followed by checking circulating lncRNAs of interest and profiling their expression using real-time PCR. Moreover, microarrays are less sensitive and specific than real-time PCR and are not reliable [47].

NGS is currently one of the best methods for detecting lncRNA, which is extremely sensitive and provides data on their relative expression in a sample with a large dynamic range compared to microarrays [48]. NGS is used to obtain small differential RNA expression profiles, including lncRNA, to study tissue developmental stages, tissue types, and pathological conditions such as oncology, with the potential to search for new biomarkers and therapeutic targets [49]. The main disadvantage of NGS is the creation of the necessary infrastructure, such as computer capacity and storage, as well as the experience of the staff to comprehensively analyze and interpret the subsequent data. In addition, you need to skillfully manage the volume of data in order to extract clinically important information into an understandable and reliable interface. The actual cost of NGS is significant. For example, current NGS technology can generate about 150,000,000 reads for about $ 1,300, while a single Sanger read typically costs less than $ 1. However, in order to make NGS cost-effective, large batches of samples would have to be run, which may require supra-regional centralization [50]. Thus, each technology has its own advantages and limitations. Therefore, the method should be selected based on the specific research requirements and the issues under consideration (Table 3) [[51], [52], [53]].

**Table 3:** Comparison of common methods for profiling lncRNAs expression. Specificity, sensitivity, flexibility, performance, and absolute quantification/accuracy are classified as follows: +++ (very high); ++ (moderate); +/++ (moderate to low); + (low); and +/− (low or not applicable). Flexibility refers to ease of setup. The only technology that allows for absolute quantification is real-time polymerase chain reaction (real-time PCR), while next-generation sequencing (NGS) is the only technology that can identify new lncRNAs. Data analysis is classified as relatively easy, moderate and complex, requiring a developed computing infrastructure. Other available problems for related technologies.

Methods	Specificity	Sensitivity	Flexibility	Productivity	Absolute quantification/accuracy	Possibility of identification of new lncRNAs	Data analysis	Problems
Real-time PCR	+++	+++	+++	+/++	+++	No	Relatively easy	Lack of consensus on data normalization
Microarray	+	+	+/−	+++	+	No	Moderate. Depends on the applications used	High price. Multiple samples on one platform
NGS	+++	++	+++	+++	++	Yes	Complicated	High price

## 3 Blood or urine?

One of the key issues in the field of research on circulating ncRNAs, including lncRNAs as biomarkers, as the choice of the priority biological fluid (urine or blood), must be resolved in order to maximize the potential of circulating lncRNAs for diagnosis, prognosis and choice of therapy for tumors of the genitourinary system. Since the blood is in contact with all-important organs (e.g, kidney), it collects all the necessary information about changes in the body. Obviously, when looking for potential biomarkers for pathologies, blood should be used. At the same time, it is available with little or no harm. The question is how long the changes can remain in the blood. In this case, it depends on how quickly the biomarker is produced and enters the bloodstream, and how quickly it leaves the blood. Blood (plasma/serum) is one of the available biological fluids for profiling the expression of circulating ncRNAs in patients with tumors [54]. Plasma or serum has been shown to be informative for profiling the expression of circulating RNA for diagnosis, prognosis, assessment of response to therapy and tumor recurrence, as well as detection of emerging resistance to therapy in various human tumors, including BC, PC, RCC [17,20,22]. The detection of circulating lncRNAs in plasma/serum samples without measurable circulating tumor cells suggests that circulating lncRNAs in plasma/serum may provide useful information about tumors of the urinary system, regardless of the presence of circulating tumor cells. Several studies have reported the sensitivity of circulating tumor cells in the blood of patients with RCC ranging from 20% to 40% [55]. However, due to the presence of a filtration barrier in the urinary system (an analogy of the blood-brain barrier for the central nervous system), blood is not an ideal fluid for an accurate assessment of these biomarkers [56]. These studies have shown conclusively that the filtration barrier can be an obstacle preventing circulating tumor cells or circulating lncRNAs from entering the bloodstream. However, as in the case of brain tumors, a violation of the barrier function occurs, which allows various molecules, including circulating for lncRNAs, to penetrate from the urinary system into the bloodstream, so it is possible from the general bloodstream into the urine.

Urine is probably the best place to detect changes, as it is a blood filtrate and contains all soluble biomarkers including circulating lncRNAs. It is possible that urine will be a better source of biomarkers than blood for diseases of the genitourinary system, including tumors [57]. Unlike blood, urine is in direct contact with the organs of the urinary system (kidney and bladder) and is a suitable source of biomarkers for tumors of this system. In addition, urine is easier and safer to obtain than blood from venipuncture or tumor tissue from biopsy. Other examples are probably saliva and salivary glands, sweat and sweat glands, cerebrospinal fluid and brain.

As already reported, EVs are membrane-enclosed nanoparticles that are released from living tumor cells, either as a result of the fusion of the endosome with the plasma membrane (exosomes) or directly from the cell membrane (microvesicles) [11]. EVs are carriers of communication between different compartments of a tumor and its microenvironment since other tumor cells and normal cells absorb them [18]. It is important to note that EVs, which can be isolated from both blood and urine, are a rich source of tumor molecules such as DNA, ncRNA, proteins, lipids, and metabolites since the EV structure protects them from nucleases and proteases [18,26]. Since blood contains a large number of nucleases and proteases, the isolation of lncRNA from EV can give a higher concentration of RNA compared to non-vesicular forms of circulating lncRNA from whole blood, plasma, or serum [13].

In addition, the change in the expression profile of circulating lncRNAs in urine and blood in the same patient with a RCC or BC is unique because urine likely reflects local events of damaged tissue compared to blood flow. Moreover, a specific panel of circulating lncRNAs in urine can help distinguish a tumor of the kidney or bladder from other inflammatory, infectious and traumatic lesions of the urinary system.

## 4 Authors' opinion

The rapidly growing number of newly discovered lncRNAs and the accumulation of experimental data explaining their multifaceted functionality promise a better understanding of tumor biology and their future use in clinical practice. However, current research aimed at comprehensively investigating lncRNAs in tumors faces several challenges. Currently, most new transcripts are discovered using NGS technologies, which face computational constraints in terms of short sequence length, mapping and de novo assembly of transcripts derived from the tumor genome with complex structural rearrangements (for example, large deletions or insertions, chromosome fusions, chromotripsis or chromoplexy).

Recent developments in fluorescence probe design, imaging and imaging technology enable the determination of (sub) cellular localization and measurement of the absolute expression of endogenous transcripts in individual cells with single molecule resolution in situ. Elucidation of the function of lncRNA is difficult due to their relatively low sequence conservatism. However, the main functionality of RNA can be found in its tertiary structure, determined by the laws of conservative sequences that promote RNA folding and are necessary for binding to proteins. This is demonstrated by the sequential assembly of Prostate cancer associated non-coding RNA 1 (PRNCR1) and lncRNA prostate cancer gene expression marker 1 (PCGEM1) with the androgen receptor (AR), the MEG3 structure required for its tumor suppressive function. Another key question that remains to be answered is what causes the strikingly specific expression of most lncRNAs in normal and tumor tissues. Genetic/epigenetic aberrations that control lncRNA expression should be investigated in the future to understand the role of lncRNA in the tumor process.

Changes seen in biological fluids usually reflect changes in tissues or cells. Theoretically, oncogenic circulating molecules, like oncogenic lncRNAs, should reflect the onset of the tumor process. However, not all tumor loci are identical. Thus, understanding the origin of these molecules, whether primary or metastatic, is a key challenge. In addition, despite the encouraging data, significant challenges remain before these tests are ready for clinical use. Both quantitative and qualitative changes in the expression of circulating lncRNA strongly depend on methodological aspects. Laboratory staff and clinicians must collaborate to address a range of issues related to consistent and feasible preanalytical and analytical conditions for selected biomarkers, as well as assay validation in well-designed and sufficiently powerful multicenter studies. It is important to describe the sampling, processing and measurement conditions as accurately as possible. The biological fluid chosen for research must also correspond to the question of interest to us. Some body fluids are more complex than others are (blood is more complex than urine), which can make analysis difficult.

Various studies have been published regarding the optimal approach to diagnosis and follow-up in patients with suspected bladder cancer. New FDA-approved tests based on detecting tumor-associated proteins, either in urine or in desquamated urothelium cells, as well as tests based on detecting chromosomal aberrations, have failed in the clinic. Several urinary lncRNA biomarkers have shown higher sensitivity and specificity than cytology, but have failed to replace invasive cystoscopy as a diagnostic standard. It can be expected that individual biomarkers or their combinations will be introduced into clinical practice in the near future, as suggested by the first results of the ongoing large prospective study of the European FP7 UROMOL project. Compared with PC and BC, there is little evidence for the diagnostic and prognostic potential of circulating serum/plasma or urine lncRNAs in patients with suspected RCC. Studies of lncRNA-based biomarkers in biological fluids remain a topic of great interest, and circulating lncRNAs have great potential for future use in medicine.

## 5 Conclusion

LncRNAs are currently considered as potential biomarkers and therapeutic targets in many pathological conditions in humans. The success of their implementation in personalized medicine will largely depend on the availability of a reference method that will effectively validate and verify reliable and specific biomarkers based on lncRNA. By focusing on certain forms of transport (eg, exosomes), improved sampling and isolation of total RNA, combined with absolute quantification without the use of housekeeping genes, may lead to the practical use of circulating lncRNAs as non-invasive biomarkers in oncourology in the future. We need to know which biological fluid of the human body (whole blood, plasma/serum, or urine) and for which pathology, in particular tumors of the genitourinary system, is best suited for measuring the expression level of circulating lncRNA. In addition, the discovery of new lncRNAs should be further confirmed by independent studies. Using a panel of two or more selected lncRNAs may be more effective and guarantee specificity for a particular pathology, such as tumors of the genitourinary system. The solution of these issues will make it possible to redirect basic research with lncRNA in oncourology to the practical application of lncRNA as therapeutic targets and biomarkers.

## Funding

The reported study was funded by RFBR and 10.13039/501100014220NSFC, project number 21-515-53017.

## Declaration of competing interest

The authors declare that no conflicts of interest exist.
